# Resting-State and Task-Based Functional Connectivity Reveal Distinct mPFC and Hippocampal Network Alterations in Major Depressive Disorder

**DOI:** 10.3390/brainsci15111133

**Published:** 2025-10-22

**Authors:** Ekaete Ekpo, Lysianne Beynel, Bruce Luber, Zhi-De Deng, Timothy J. Strauman, Sarah H. Lisanby

**Affiliations:** 1 Noninvasive Neuromodulation Unit, Experimental Therapeutics & Pathophysiology Branch, National Institute of Mental Health, Bethesda, MD 20892, USA; eekpo1@alumni.jh.edu (E.E.); bruce.luber@nih.gov (B.L.); zhi-de.deng@nih.gov (Z.-D.D.); 2 Adult Psychiatry & Psychology Division, Department of Psychiatry & Behavioral Sciences, Duke University School of Medicine, Durham, NC 27705, USA; tjstraum@duke.edu; 3 School of Medicine and Advanced Medical Engineering, Arizona State University, Tempe, AZ 85258, USA; holly.lisanby@asu.edu

**Keywords:** functional connectivity, fMRI, depression, goal-priming task, resting state

## Abstract

**Background:** Resting-state functional connectivity (RSFC) is widely used to identify abnormal brain function associated with depression. Resting-state functional magnetic resonance imaging (fMRI) scans have many potential confounds, and task-based FC might provide complementary information leading to better insight on brain function. **Methods:** We used MATLAB’s (version 2024b) CONN toolbox (version 22a) to evaluate FC in 40 adults with and without major depressive disorder (MDD) (n_MDD_ = 23, n_HC_ = 17). fMRI acquisition was performed while participants were at rest and while performing the Selves Task, an individualized goal priming task. Seed-based analyses were performed using two seeds: medial prefrontal cortex (mPFC) and left hippocampus. **Results:** Both groups showed strong positive RSFC between the mPFC and other DMN regions, including the anterior cingulate cortex and precuneus, which had more focal positive FC to the mPFC during the task in both groups. Additionally, the MDD group had significantly lower RSFC between the mPFC and several regions, including the right inferior temporal gyrus. The left hippocampus seed-based analysis revealed a pattern of hypoconnectivity to multiple brain regions in MDD, including the cerebellum, which was present at rest and during the task. **Conclusions:** Our results indicated multiple FC differences between adults with and without MDD, as well as distinct FC patterns and contrast results in resting state and task-based analyses, including differential FC between mPFC–cerebellum and hippocampus–cerebellum. These results emphasize that resting-state and task-based fMRI capture distinct patterns of brain connectivity. Further investigation into combining resting-state and task-based FC could inform future neuroimaging research.

## 1. Introduction

Major depressive disorder (MDD) affects about 300 million people globally [1]. Depressive symptoms can severely impair functioning and are often associated with abnormal brain function [2,3,4]. Neuroimaging is a powerful tool to investigate differences between people with and without MDD and therefore represents a way to better understand this disorder and potentially develop new treatment approaches for patients. Functional connectivity (FC), which is assessed by correlating blood oxygen level-dependent (BOLD) timeseries between brain regions [5,6], is widely used to identify abnormal brain function associated with psychiatric disorders, including MDD [2,3,7].

Resting-state fMRI (RS fMRI) and resting-state FC (RSFC), during which participants are asked to rest and/or stare at a fixation point, are the most commonly used paradigms to investigate differences between patients with MDD and participants without this disorder (e.g., [6,8]). RSFC studies have informed several RS network theories on the pathology of MDD (e.g., [3,9]). These theories postulate dysfunction within and between several networks, such as the affective network, default mode network (DMN), central executive network (CEN), and salience network (SN) in MDD symptomatology. These theories are supported by RSFC network findings, which indicate reliable RSFC patterns, such as strong positive FC within the DMN [2,3,7,10,11,12] and negative FC between the CEN and the DMN [7,13]. For participants with MDD, multiple studies have indicated aberrant RSFC between the DMN, CEN, and SN [14,15,16]. Studies comparing FC in participants with and without depression have indicated higher positive within-network FC in the DMN of people with depression [2,3,10,11,12,17]. Interestingly, hyperconnectivity within the DMN has been linked to rumination, which is believed to be an important factor in the pathology of depression [18,19,20,21].

Although RSFC studies have resulted in a fairly consistent set of observations that inform current theories of depression-related brain networks, this approach suffers from many potential confounds that complicate analyses and their interpretation. For example, variability in emotions and unrelated thought processes, such as introspection, during acquisition could bias the results [8]. A potential way to address this limitation is to analyze both RSFC and task-based connectivity in order to obtain complementary information. While there are as yet only a small number of direct comparisons of these two techniques, emerging evidence suggests that collecting and analyzing both types of information could provide convergent evidence (e.g., in spatial overlaps between the two measures) (e.g., [22,23]), or might reveal connectivity evoked by performance of the task that might be too weak to appear in a resting measure [24], providing more specific insights into brain-behavior relationships [25,26]. Therefore, in this study, we used task-based FC in combination with RSFC to elucidate FC among depression-related brain networks and observed the similarities and differences found with each measure. Specifically, for task-based FC, we employed the Selves Task, which has previously been successfully used in imaging studies to examine differences between patients with MDD and healthy controls [27,28,29,30].

The Selves Task is a goal priming fMRI task derived from the Selves Questionnaire [27,31], which involves the subliminal presentation of words related to the personal goals provided by the participant. In this task, promotion goals, which represent a person’s aspirations and ideals, or what they hope to achieve to make good things happen—are contrasted with prevention goals, which represent duties and obligations, or what a person feels they should do to prevent bad things from happening. This task has been shown to evoke activity related to promotion-based and prevention-based thinking about goal pursuit in brain networks related to emotional regulation that may be dysfunctional in MDD [27,28,29,30]. It also highlights differences in brain activation patterns between healthy controls and people with MDD on brain activations [28]. Interestingly, another study from our group demonstrated that FC was also associated with differences between these two groups during promotion and prevention priming, including a higher FC within the DMN and limbic system for people with MDD [32]. However, this study used a small sample size and a correlational psychophysiological interaction (PPI) analysis, which, while informative about connectivity patterns, did not rely on a singular seed region but instead assessed correlations across the whole brain. Seed-based studies using individual brain regions can provide answers to specific research questions, allowing for clearer interpretations of results [33]. Thus, in the current study, we aimed to expand on the Davis et al. (2023) findings [32], including DMN hyperconnectivity using a larger sample size and with a seed-based FC analysis to offer a more hypothesis-driven approach [30,34].

While previous fMRI work with the Selves Task focused on a distributed brain network related to emotional regulation, in the present study we chose to investigate fMRI activations due to regions involved with self-referential processing and with emotion/memory integration. These are processes the Selves Task requires, and which may also show illuminating differences between healthy adults and those with depression. For our first seed, we selected the medial prefrontal cortex (mPFC) given its central role within the DMN, and because several studies demonstrated its strong involvement in self-referential processing, a cognitive process involved in the Selves task. For example, studies demonstrated that when healthy volunteers were presented with positive and negative personality traits and asked to either judge whether these words described them, or to simply judge whether they described a desirable trait, activations within the mPFC was specific to self-referential judgment [35]. Interestingly, other studies investigating differences between healthy volunteers and participants with MDD demonstrated aberrant activation of the mPFC in patients, only during self-referential judgment (e.g., [36,37]). Consequently, we expected that task-based FC analysis, centered on prevention goal priming would show FC differences between MDD and HC within the DMN. On the other hand, the RSFC analysis would indicate more extensive FC between the mPFC and the rest of the DMN, and possibly even more positive FC for participants with MDD compared to HCs, given the higher levels of rumination in MDD [38], and which would replicate previous results [18,19,20,21,39].

Given the relevance of goals to an individual’s declarative memory system, another aspect of the Selves task that has not yet been explored is its potential to elucidate network dysfunction in MDD associated with the hippocampus, a region critically involved in memory and emotion processing. Studies have demonstrated that semantic word priming can induce hippocampal activation [40,41], supporting the idea that using a hippocampus seed might reveal connectivity patterns involved in MDD as yet unreported. Given the use of verbal stimuli (familiar words) in the Selves task, the left hippocampus, a key contributor to memory and emotion integration [42], was selected as our seed of interest. Prior studies using similar subliminal presentation of emotionally salient words demonstrated activations in emotion-related regions such as the amygdala and fusiform gyrus [43,44,45,46,47]. Building on these findings, we expected that the promotion and prevention goal words—generated by participants—would induce FC between the left hippocampus and emotion-processing regions such as the amygdala, with differences in FC in MDD compared to HC. On the other hand, studies of resting-state fMRI have investigated hippocampus connectivity and demonstrated positive FC with several regions within medial-temporal network, such as the right hippocampus, and parahippocampal gyri, as well as the DMN in patients with MDD and in healthy controls [48,49,50], and differences between MDD and HC groups in the prefrontal cortex, parietal cortex and cerebellum [49]. In our resting state analysis, we therefore expected to also find positive connectivity between the left hippocampus and these regions in both groups, and some differences between MDD and HC.

## 2. Materials and Methods

### 2.1. Participants

Participants included adults with MDD (MDD group, n = 23, mean age = 43, SD_age_ = 14 years, 61% female) and adults without psychiatric disorders (HC group, n = 17, mean age = 33, SD_age_ = 12 years, 47% female). All participants provided written informed consent prior to participation.

Participants in the MDD group were enrolled in a clinical trial investigating the use of concurrent fMRI-guided repetitive transcranial magnetic stimulation (rTMS) and cognitive therapy for the treatment of major depressive episodes (NCT03289923). They had no Axis I comorbidities besides generalized anxiety disorder. They all had at least one failed antidepressant trial and had scores within our pre-determined thresholds for the Hamilton Depression Rating Scale (HDRS), Montgomery–Åsberg Depression Rating (MADRS), and Young Mania Rating Scale (YMRS) [HDRS ≥ 17 or MADRS ≥ 20, and YMRS < 12]. For the HC group, participants with a personal history of psychiatric diagnoses and/or a history of psychiatric diagnoses in their immediate families were excluded.

All participants underwent mental health evaluations using the Structured Clinical Interviews for Diagnostic and Statistical Manual of Mental Disorders, 4th edition (DSM-IV) (SCID) to ensure that they met the diagnostic criteria for their group. Participants with hearing loss, serious neurological conditions, and severe unmanaged physiological conditions were excluded from both groups. Table 1 provides the demographic data for each group. There was a significant group difference in age [t(38) = −2.417, *p* = 0.021], but no significant group difference between their sexes [*χ*^2^(1) = 0.753, *p* = 0.385]. While we found a group difference in age, we did not include this variable into the fMRI analysis, as previous research from Strauman et al. has failed to reveal substantial age differences in adults age 18–65 in response to promotion or prevention goal priming [29].

### 2.2. Procedure

After consenting to the protocol, all participants completed the event reaction questionnaire (ERQ), which consists of Likert scale questions with five response options ranging from certainly false (1) to certainly true (5). The ERQ has six promotion-related questions and six prevention-related questions. Responses to these 12 questions were scored to yield four subscale scores (Promotion History, Prevention History, Promotion Success, and Prevention Success). Promotion History indicates how much the respondent believes their parents focused on making good things happen. Prevention History indicates how much the respondent believes their parents focused on keeping bad things from happening. Promotion Success indicates how successful the respondent believes they have been at making good things happen in their lives. Prevention Success indicates how successful the respondent believes they have been at keeping bad things from happening in their lives. The ERQ was administered to confirm the validity of the fundamental differences between promotion and prevention focus in our cohort of participants, and we expected the HC group to have higher scores in all subscales, given that MDD is known to be associated with negative self-perception [51,52]. Furthermore, we investigated whether ERQ scores would correlate with FC differences between participants with MDD and the HC group.

Then, participants completed the Selves Questionnaire [31,53], which was used to generate stimuli for the fMRI task. Participants were asked to provide one-word responses to three questions: (1) What are the attributes that you believe you actually possess? (2) What are the attributes of the kind of person that you would ideally like to be? (3) What are the attributes of the kind of person you believe you ought to be? Responses to these questions were categorized as follows: (1) Actual, (2) Ideal, and (3) Ought. Word-pairs between these three lists were further categorized as follows: (A) synonyms between the ideal and actual list (Promotion Match), (B) antonyms between the ideal and actual list (Promotion Mismatch), (C) synonyms between the ought and actual list (Prevention Match), (D) antonyms between the ought and actual list (Prevention Mismatch). Promotion Match and Promotion Mismatch words indicated concordance and discordance, respectively, between one’s actual self-perception and one’s ideal self (successful and unsuccessful promotion-based goal pursuit, respectively). Similarly, Prevention Match and Prevention Mismatch words indicated concordance and discordance, respectively, between one’s actual self-perception and one’s ought self (successful and unsuccessful prevention-based goal pursuit, respectively). When selecting words to include in the fMRI stimuli for each participant, we did not include words that appeared in two or more of the three lists that were same of synonymous.

Urine pregnancy tests were performed prior to scanning, and participants underwent structural and functional MRI acquisition. Participants were then asked to return for a second MRI session approximately six weeks after the first, to mirror the time interval of the MDD group who received six weeks of rTMS before their second MRI scan. Only the baseline MRI data are included in the current work.

### 2.3. Selves Task Stimuli

**Figure 1:**
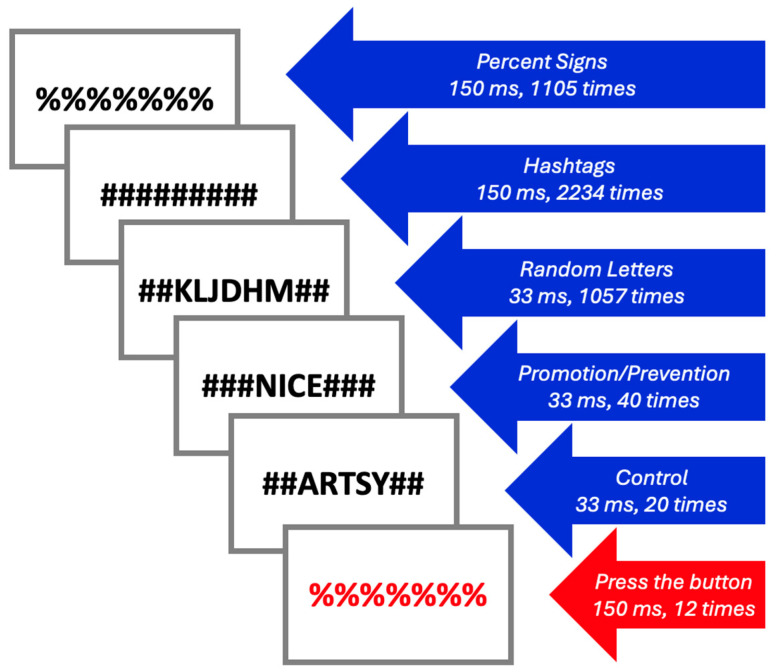
Selves Task stimuli.

Each fMRI stimulus block contained two words from the participants’ Selves questionnaire responses (Promotion and Prevention), two semantically unrelated words from the responses of a random HC (Control), as well as random strings of letters, hashtags (#), and percent signs (%) to allow for visual masking (Figure 1). To ensure that participants were engaged throughout the task, participants were asked to press a red button when they saw red text. Each fMRI block lasted 9 min and 15 s and included over 4000 stimuli. During each block of fMRI acquisition, Promotion/Prevention and Control words were subliminally presented for a duration of 33 ms (40 times and 20 times per block, respectively), along with hashtags (150 ms, 2234 times per block), black percent signs (150 ms, 1105 times per block), random letters (33 ms, 1057 times per block), and red percent signs (150 ms, 12 times per block). The order of the blocks was randomized.

### 2.4. MRI Acquisition

A 3T General Electric MRI scanner was used, with a standard 32-channel head coil for all acquisitions. For each participant, a T1 image was acquired (TI = 1100 ms, voxel size = 1 mm, FOV = 28 cm), followed by a 10-min resting-state scan during which participants were asked to stare at a fixation cross (TR = 3 s, TE = 16.9 ms, voxel size = 3.4 mm, FOV = 22 cm). fMRI was acquired while participants were performing the four blocks of the Selves Task (9 min, 15 s; TR = 3 s; TE = 30 ms; voxel size = 3 mm; FOV = 28 cm). DTI and T2 were also collected but not analyzed in the current manuscript. The stimuli were back projected onto a screen located at the foot of the MRI bed using an LCD projector. Subjects viewed the screen via a mirror system located in the head coil, and participants with nearsightedness and farsightedness wore MRI-safe glasses. The start of each run was electronically synchronized with the MRI acquisition computer.

### 2.5. Analysis

**Figure 2:**
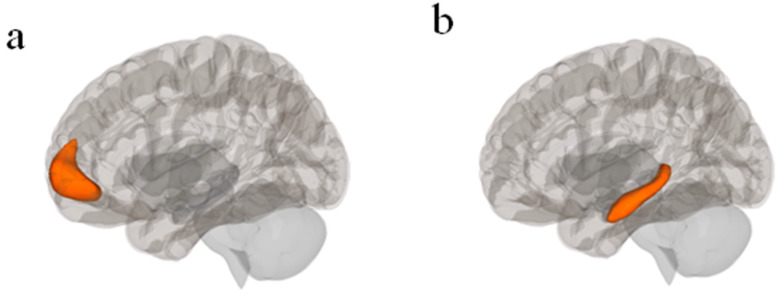
(**a**) mPFC seed in CONN Toolbox atlas with central coordinate [+1, +55, −3] (Nieto-Castanon & Whitfield-Gabrieli, S., 2022); (**b**) Left hippocampus seed in CONN Toolbox atlas with central coordinate [−25, −23, −14]; [54].

The CONN toolbox (Version 22a) was used to perform seed-based FC analyses [54]. PPI FC analyses were used for task-based FC. The left hippocampus was the seed for the Promotion + Prevention > Control contrast, and the mPFC as the seed for the Prevention Match condition. Both seeds were used for the resting state analyses as well, to allow for qualitative comparisons between the two sets of analyses. The seeds were pre-defined by the CONN toolbox atlas (default coordinates: mPFC: [+1, +55, −3]; left hippocampus: [−25, −23, −14]) (Figure 2). The analyses yielded clusters of voxels with significant FC to each seed (or no clusters, if no clusters had significant FC to the seed). Bandpass filtration was not used, to avoid excluding large amounts of functional data. False discovery rate (FDR) correction was applied to all cluster threshold *p*-values. FDR correction was applied to voxel threshold *p*-values for individual analyses but not contrasts. T-statistic thresholds [denoted as t(n) > …] indicate the lowest T-statistic of clusters allowed to appear in the analysis results. T-statistics for individual clusters [denoted as t(n) = …] indicate the average FC within each cluster. We used the default CONN toolbox processing steps in this analysis, including skull-stripping, alignment, and smoothing.

ERQ analysis was performed using JASP Version 0.19.3 [55]. A one-tailed independent samples *t*-test was performed on each of the four ERQ subscale scores.

## 3. Results

In these sections, we first present the results from the resting state analyses followed by the results from the Selves Task. For each of them, we will present results from our two seeds of interest: the mPFC and the hippocampus. For each result, we highlight the largest positive and negative clusters for each group separately, followed by a comparison between the MDD and HC groups, as well as a figure and a table showing all significant clusters.

### 3.1. Resting-State FC

Resting-state MRI data was not available for one participant in the MDD group and one participant in the HC group; therefore, the analysis was performed on 38 participants (nMDD = 22, nHC = 16). We present below the results associated with the mPFC seed, followed by the hippocampus seed.

#### 3.1.1. mPFC Seed

**Figure 3:**
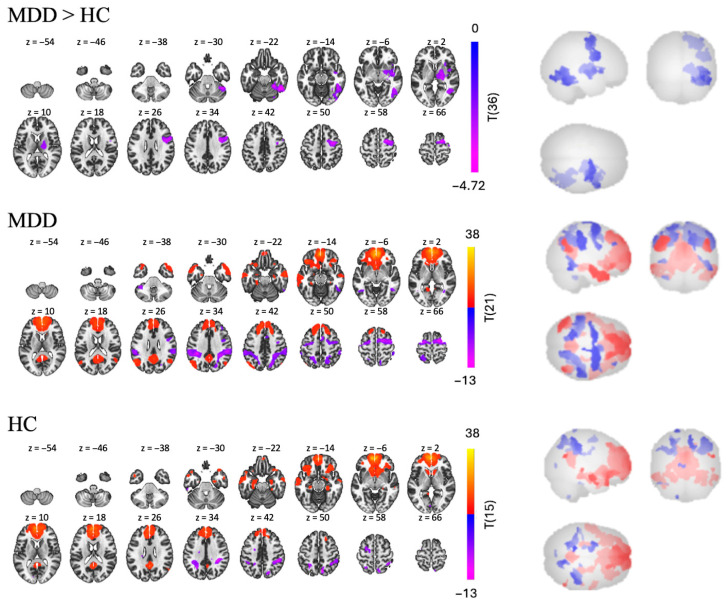
RSFC using the mPFC as a seed for MDD > HC, MDD, and HC group independently.

In the MDD group, mPFC seed-based RSFC analysis revealed 15 significant clusters, eight of which were positive and seven of which were negative [t(21) > 4.98, *p* < 0.001; see Table 2 and Figure 3]. The largest positive cluster (17,085 voxels) was in the frontal lobe and included the left and right frontal pole (lFP, rFP), left superior frontal gyrus (lSFG), and anterior cingulate cortex (ACC). Regions in the second largest positive cluster were more posterior and included precuneus, posterior cingulate cortex (PCC), and left lingual gyrus (lLG). As expected, most of these regions are part of the DMN. Regions in the largest negative cluster (3570 voxels) included the right precentral gyrus (rPreCG), right and left superior frontal gyrus (rSFG, lSFG), and left precentral gyrus (lPreCG). Regions in the second largest negative cluster (2952 voxels) included right superior supramarginal gyrus (rsSMG), right posterior supramarginal gyrus (rpSMG), right lateral occipital cortex (rLOC), and right superior parietal lobule (rSPL). Most of the regions were found to belong to “active” networks such as the fronto-parietal and dorsal attention networks.

Similar patterns were found in the HC group, in which we found 16 clusters—six positive and 10 negative [t(15) > 5.77, *p* < 0.001]. Regions in the largest positive cluster (14,764 voxels) included the rFP, lFP, ACC, and right and left paracingulate gyri (rParaCG, lParaCG). Regions in the second largest positive cluster (1143 voxels) included the PCC and precuneus. The positive clusters were found to belong to the DMN. Regions in the largest negative cluster (1012 voxels) included the rpSMG, rSPL, rSMG, and right angular gyrus (rAG). Regions in the second largest negative cluster (767 voxels) included the lSPL, laSMG, lpSMG, and sLOC, in which are part of the “active” network.

Interestingly, while the RSFC pattern of both groups were similar, with positive FC from mPFC to DMN and negative FC from mPFC to active networks, the MDD > HC contrast still revealed three negative clusters [t(36) > 2.03, *p* < 0.05], suggesting that the MDD group had hypoconnectivity compared to the HC group in some regions. Regions in the largest cluster (2382 voxels) included the right inferior temporal gyrus (rITG), rLOC and right temporal occipital fusiform cortex (rTOFusC). The second largest cluster (2313 voxels) was more frontal and included the rPreCG, rSFG, and rMidFG. Finally, regions in the smallest cluster (1951 voxels) were deeper brain structures, including the right insular cortex (rInsC), right thalamus, and right pallidum.

#### 3.1.2. Left Hippocampus Seed

**Figure 4:**
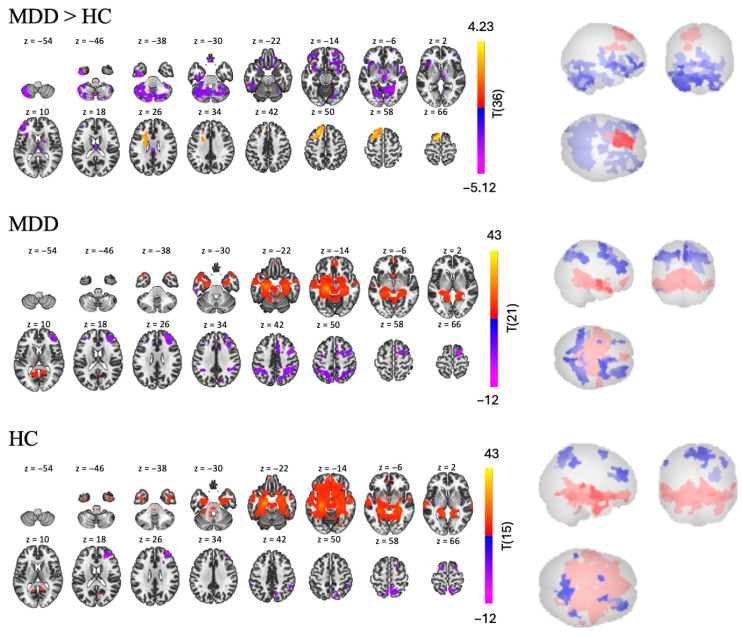
RSFC using the left hippocampus as a seed for MDD > HC, MDD, and HC groups.

In the MDD group, we found nine clusters of RSFC between the left hippocampus and other regions—two positive and seven negative [t(21) > 5.14, *p* < 0.001, see Table 3 and Figure 4]. Regions in the largest positive cluster (14,408 voxels) included the left and right hippocampus, brainstem, and left cerebellum. Regions in the largest negative cluster (2147 voxels) included the rSFG, right middle frontal gyrus (rMidFG), and rParaCG.

In the HC group, we found nine clusters of RSFC between the left hippocampus and other regions—three positive and six negative [t(15) > 5.66, *p* < 0.001, see Table 3 and Figure 4]. Regions in the largest positive cluster (21,168 voxels) included the lOFC, brainstem, left and right hippocampus. Regions in the largest negative cluster (1381 voxels) included the precuneus, right and left LOC.

The MDD > HC contrast revealed three clusters of significantly different FC between the two groups—one positive and two negative [t(36) > 2.03, *p* < 0.05, see Table 3 and Figure 4]. Participants with MDD show lower RSFC than participants in the HC group in the bilateral cerebellum and rSFG (largest negative cluster, with 9858 voxels); and in the bilateral frontal pole and right orbital frontal cortex (rOFC) (second largest negative cluster with 5054 voxels). However, participants with MDD also display higher RSFC than the HC group in a smaller cluster (2481 voxels), including left frontal regions such as the lSFG, MidFG, and lFP.

### 3.2. Task-Based Analyses Using the ERQ and the Selves Task

In the next sections, we will present the results related to the Selves Task, starting with behavioral results from the ERQ task, an indicator of promotion and prevention focus, followed by the FC results for our two seeds (mPFC and left hippocampus).

#### 3.2.1. ERQ

**Figure 5:**
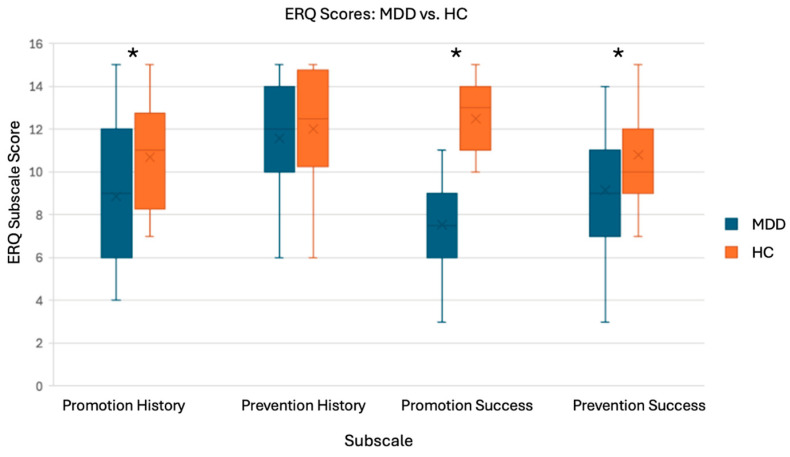
T-test results and boxplots for ERQ analysis. Subscales: Promotion History (n_MDD_ = 19, n_HC_ = 17), Promotion Success (n_MDD_ = 20, n_HC_ = 16), Prevention History (n_MDD_ = 20, n_HC_ = 17), and Prevention Success (n_MDD_ = 19, n_HC_ = 16). * There were significant group differences for all subscales, except Prevention History.

Some participants left some questions unanswered; therefore, subscales with incomplete responses were excluded from ERQ analysis. The results of the one-tailed *t*-tests on the ERQ scores were generally in line with our hypotheses as the HC group had significantly higher Promotion History, Promotion Success, and Prevention Success scores, compared to the MDD group [t(34) = 2.016, *p* = 0.026; t(34) = 8.385, *p* < 0.001; t(33) = 1.980, *p* = 0.028). For the Prevention History subscale, there was no significant group difference [t(33) = 0.70, *p* = 0.245]. These results suggest that our two groups have different promotion and prevention focuses (Figure 5) and support the validity of our use of promotion- and prevention-related words to elicit differences between groups with the Selves Task.

#### 3.2.2. Task-Based FC: Selves Task

As one participant in the HC group was unable to see the stimuli, one participant in the HC group did not undergo task-based fMRI, and one participant in the MDD group was presented with the wrong stimuli, the task-based FC analyses were performed on 37 participants (n_MDD_ = 22, n_HC_ = 15). We will first present the results from the mPFC seed, followed by the results from the hippocampus seed.

##### mPFC Seed (Prevention Match Stimuli)

For this seed, we examined the Prevention Match condition, as we expected prevention goal priming to evoke negative thoughts/feelings and thus more positive FC between the mPFC and the DMN. Contrary to our expectations, we did not find any significant differences between the MDD and HC groups; therefore, we only present the results from each group individually below.

**Figure 6:**
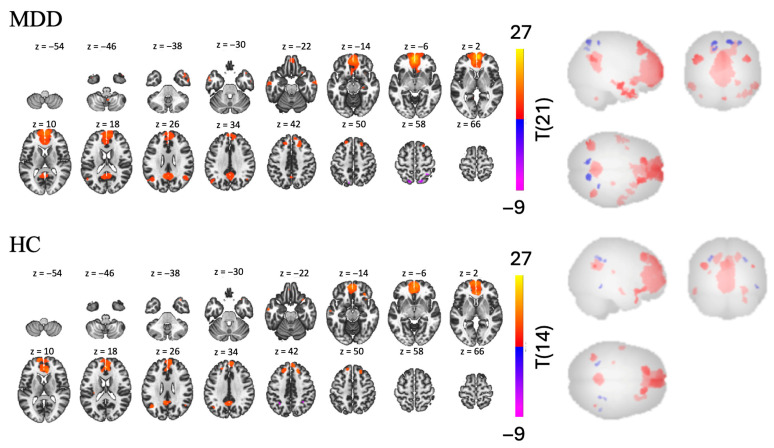
Task-based FC in the Prevention Match condition using the mPFC as a seed for MDD and HC groups.

In the MDD group, we found 17 significant clusters, 13 had positive FC to the mPFC and had negative FC to the mPFC [t(21) > 5.48, *p* < 0.001, see Table 4 and Figure 6]. Regions in the largest positive cluster (7237 voxels) were in frontal areas, including left and right frontal pole (rFP, lFP), ACC, left and right ParaCG. Regions in the second largest positive cluster (1488 voxels) were more posterior and included the precuneus and PCC. The two largest negative clusters were way smaller and included the right and left LOC with respectively 90 and 53 voxels.

In the HC group, we found 13 clusters of FC between the mPFC and other regions—9 positive and 4 negative [t(14) > 6.62, *p* < 0.001, see Table 4 and Figure 6]. Regions in the largest positive cluster (4685 voxels) included the AC, left and right frontal pole, right and left ParaCG. Regions in the second largest positive cluster (446 voxels) included the PCC and precuneus. Similarly, the negative clusters were very small (37 and 19 voxels), and the two largest included the lSPL, lpSMG, and rSPL.

##### Hippocampus Seed (Contrast: Promotion and Prevention Words > Control Words)

**Figure 7:**
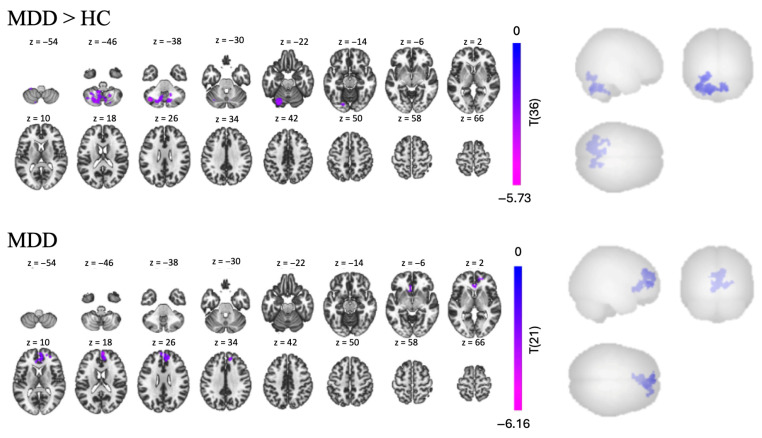
Task-based FC in the Promotion + Prevention > Control contrast, using the left hippocampus as a seed for MDD > HC comparison and MDD group individually.

Since the Promotion and Prevention trials contained stimuli generated by the participants that were related to their own goals, we hypothesized that they would evoke subconscious memory. Therefore, we examined a contrast comparing FC from all familiar words vs. all unfamiliar words: the Promotion + Prevention > Control contrast. In the MDD group, we found one cluster of negative FC, with 1447 voxels, between the hippocampus and other regions, including the rParaCG, ACC, rlFP, SubCalC, and lFP [t(14) > 2.14, *p* < 0.05, see Table 5 and Figure 7]. Contrary to our assumption, we did not find any significant clusters in the HC group. However, the MDD > HC contrast revealed one significant cluster of lower FC in the MDD group, predominantly occupied by several regions of the cerebellum.

## 4. Discussion

In this study, we compared results from resting-state and task-based FC in patients with MDD vs. HC. We were expecting our analysis to reveal both spatial overlaps between resting-state and task-based functional connectivity, as well as unique pattern specific to each. We focused on two seeds the mPFC and the left hippocampus relevant to self-referential processing and memory/emotion integration, respectively. The mPFC seed revealed a strong positive connectivity with the DMN at rest, which became more constrained to specific regions of the DMN during the task. Interestingly, when focusing on the hippocampus seed, we found a pattern of hypoconnectivity in patients with MDD compared to HC between the left hippocampus and the cerebellum that was present but at rest, but became more pronounced during the task, involving more regions of the cerebellum. These findings suggest that rest and task fMRI offer complementary perspectives on functional connectivity in MDD. Resting measures revealed intrinsic network organization, while the task, eliciting self-referential content, unmasks condition-specific disruptions. The hippocampus–cerebellum circuit, identified across both modalities, may represent a convergent network alteration with relevance for understanding memory-emotion integration in depression, with potential as a treatment target.

### 4.1. mPFC Findings

Using the mPFC as a seed, we expected to find task-based differences between HC and MDD in specific regions of the DMN (precuneus and PCC); and a more widespread positive RSFC with the entire DMN that would be even stronger in MDD compared to HC.

#### 4.1.1. Resting-State FC

As expected, we found in both groups a strong and widespread positive FC between the mPFC and other DMN regions. Results also revealed negative FC with regions associated with externally directed, task-positive networks such as the dorsal attention and the frontoparietal networks (e.g., SPL, SFG). These findings are consistent with the mPFC’s central role in self-referential processing [13], its key role in rumination [19,37,56], and its anti-correlational relationship with externally directed networks [13].

However, contrary to our expectations, when comparing both groups, we did not find higher FC from the mPFC to the rest of the DMN or any other regions in the MDD group compared to HCs. This contradicts literature that has indicated differential within-DMN FC in MDD compared to healthy controls [2,3,10,11,12,17,57,58,59], including mPFC seed-based studies indicating this same pattern [60], along with mPFC-SN connectivity [61]. While higher connectivity within the DMN in patients with MDD has been a common finding associated with resting state analyses, there have also been reports that they could also display lower FC compared to HC. For example, Williams (2017) [38] mentioned that several studies found hypoconnectivity of the DMN in patients with depression that could be related to impairments in self-reflection. Analysis of data from the REST-meta-MDD Consortium which included more than 1000 MDD patients and more than 1000 HC, showed that DMN hypoconnectivity was characteristic in patients with recurrent MDD [62]. Given that our MDD group included only treatment-resistant MDD patients, it is possible that our results are specific to this population. Tozzi et al. (2021) [63] used the same data set as Yan et al. (2019) [62] and investigated RSFC by splitting the DMN into three subsystems. They also found lower connectivity in patients compared to healthy controls in the DMN core (mPFC–PCC) but highlighted the large variability across the sample. This suggests that further research on how depression severity, concurrent medications, and demographics (like race and age) affect DMN connectivity could provide insight on variations in DMN/MDD findings.

What the comparison of MDD and HC in our sample did show were three clusters of significantly lower FC (hypoconnectivity). The first cluster contained temporal regions such as the right ITG and several cerebellar areas. The right ITG plays a role in semantic memory, which may be deficient in MDD [64,65,66]. This result also aligns with deficiencies in working memory and sensory relay in people with MDD [67,68]. The second cluster was more frontal, centered in the right hemisphere, and included regions such as the precentral gyrus, SFG and MidFG. Hypoconnectivity between the right dlPFC/right dmPFC and the rest of the brain has been linked to treatment-resistant depression [69]. Thus, deficient FC between the mPFC and dlPFC may indicate dysfunction in how both regions communicate to the rest of the brain. The last cluster of hypoconnectivity included subcortical structures such as the insula, thalamus, and pallidum. Interestingly, the insula is a key node of the SN, and this result might reflect disrupted interactions between the SN and DMN. Furthermore, a recent RSFC study indicated decreased FC between the ITG and the SN in people with MDD, compared to HCs [65]. Findings like these give credence to the theory of deficient inter-network communication posited by Triple Network Model [3,4]. Interestingly, although the thalamus is highly connected to the salience network and the pallidum is part of the basal ganglia, neither structure is considered a core node of the SN. All these regions (prefrontal cortex, thalamus, and basal ganglia) have been suggested to form the neuroanatomical network of mood regulation [70] and have been implicated in emotion regulation and the pathophysiology of depression (e.g., [38,71]).

#### 4.1.2. Selves Task FC

As expected, the task-based connectivity approach revealed a more spatially restricted involvement of DMN regions compared to the resting state analysis. In both groups, we observed a positive cluster in frontal areas including the frontal pole, anterior cingulate cortex (ACC), and paracingulate cortex. The second cluster in each group encompassed posterior regions, including the precuneus (in MDD) and PCC (in HC). These findings align with our hypothesis that prevention goal priming would elicit positive connectivity with posterior DMN regions, including the precuneus and PCC, both involved in self-referential processing [72]. In addition to positive FC between the mPFC, PCC, and ACC, the prevention goal priming engaged adjacent frontal regions such as the frontal pole and paracingulate cortex. Interestingly, those two regions have been found to be activated during the Selves task, but in the context of promotion goal priming [29] and not prevention. It is possible that the changes in BOLD activity do not transfer directly to changes in FC.

Contrary to expectations, we did not find any significant differences between the groups. We were expecting that, because of heightened thoughts of avoidance, the MDD group would have higher within-network FC in the DMN during the prevention-related trials of the task, compared to the HC group, as these goals could induce ruminative thoughts. This result was surprising because prevention was associated with BOLD activation and connectivity differences in previous studies as assessed by ICA and whole brain PPI analyses [28,30,32]. While prevention goal priming can induce changes captured by ICA, it may not have been strong enough to be captured by our seed-based PPI analysis. It is also possible that using the mPFC as a seed was not the best choice to observe this difference, as Strauman et al. 2013 [29] indicated differences in the precuneus and PCC.

### 4.2. Findings with Left Hippocampus Seed

We expected to find differences in FC in MDD compared to HC between the left hippocampus and emotion-processing regions such as the amygdala and prefrontal emotional regulation and goal planning regions (e.g., Helm, 2018 [73]) during the presentation of promotion and prevention words compared to control words. In addition, following the results from Cao et al. (2012) [49] we expected positive connectivity centered around parahippocampal regions in both groups during resting state, with some differences between MDD and HC groups in the prefrontal cortex, parietal cortex and cerebellum.

#### 4.2.1. Resting-State FC

In both groups, we found large and robust positive FC between the hippocampus and several areas of the limbic system, which is consistent with past literature indicating positive FC between the left hippocampus, right hippocampus, parahippocampal gyri, and DMN in patients with MDD, as well as in healthy participants [48,49]. Similarly to Cao et al. (2012) [49], we also found clusters of negative FC between the hippocampus and the right prefrontal cortex, and the precuneus (predominantly in HC), which as discussed in Cao et al. (2012) [49] are rarely reported in the literature and need to be further explored.

While we did not find group differences in the left hippocampus and parietal cortex, similarly to Cao et al. (2012) [49] we found a cluster of positive FC to the left prefrontal cortex (SFG, and MFG) that encompassed the left dlPFC, which is highly involved in depression (e.g., [74]). This result, combined with what was found using the mPFC as a seed, point towards an interesting pattern of lateralization when comparing both groups, with hypoconnectivity between the mPFC and the right PFC, and hyperconnectivity of the hippocampus with the left prefrontal cortex in patients compared to healthy controls. This pattern may reflect an imbalance in hemispheric contributions to emotional regulation and memory integration, potentially underlying the cognitive and affective symptoms observed in depression [75,76,77]. We also found lower FC for patients with MDD compared to HC within the bilateral frontal pole, but the largest hypoconnectivity, was also found in the cerebellum, similarly to Cao et al. (2012) [49]. This result will be further discussed below.

#### 4.2.2. Selves Task: Promotion + Prevention > Control

In the task-based analysis, we found that using individualized goal words evoked hypoconnectivity in patients compared to HC between the left hippocampus and both regions associated with emotion processing such as ACC and paracingulate gyrus and with goal planning such as the frontal pole. These results suggest a potential downregulatory relationship in MDD between memories, emotions and plans tied to the individually familiar goal words of the Selves Task, and could be linked to dysfunction between cognition, emotion, and memory—a complex but common issue underlying depression [78]. On the other hand, we did not find significant connectivity between the hippocampus and any other brain regions in the HC group when presented with personalized words compared to control words. One possibility was that the control words failed to produce a group FC difference due to similarity between participants’ words and the control words used.

When comparing both groups, we found the strongest cluster of significant hypoconnectivity in patients with MDD compared to HC between the left hippocampus and the cerebellum, including cerebellar lobules VI and VII and Crus I and II, all areas associated with cognition and language [79]. As mentioned above, this [80] was also true using the left hippocampus seed in the RSFC analysis, although the result involved more regions of the cerebellum in the task-based analysis. There is a growing literature concerning interactions between the hippocampus and the cerebellum at both physiological and behavioral levels [81,82]. As with cerebro-cerebellar connectivity, hippocampal-cerebellar anatomical connections are multi-synaptic and numerous [83]. Coordinated activity between the two structures has been shown using electrophysiological [84] and optogenetic measures [85,86]. Certain time-sensitive kinds of conditioned learning have been shown to require coordination between the cerebellum and the hippocampus [87], as does the learning of some spatial tasks in rodents [88,89] and humans [90].The degree to which hippocampus and cerebellum have been shown to act together as a functional system in the brain has even inspired a name for that system: the “hippobellum” [81].

As has been pointed out in a more general context, the mode of action for antidepressants might be in normalizing interactions of brain networks rather than by righting neurochemical imbalances (e.g., [73]). While the hippocampus is known to be involved in depression (e.g., [91]), evidence is also growing for involvement of the cerebellum as well- that it plays a large role in the regulation of cognitive and affective processing, that damage to it leads to deficits in those processes [79,92], and that it specifically is involved in depression [93]. In this light, findings in this study and in others of altered functional connectivity between depressed patients and healthy volunteers within the hippobellum network take on some significance [49,94,95,96,97].

Studies of the brain networks involved in depression (including our own) tend to focus on the cerebral cortex, notably DMN, SN, and CEN; however, dysfunction in the interaction of subcortical structures and the cerebral cortex may also be important both in the study of depression and in potentially providing biomarkers to identify different subgroups within this heterogeneous disease (e.g., [98,99]). Of note, more work needs to be done if FC between hippocampus and cerebellum can be used as a potential biomarker, given that in the results to date, two studies [49,96] show hyperactivity between the two structures in depressed patients and two others including our own show hypoactivity [97,100]. Seed choice in FC analyses also needs greater exploration: while using the hippocampus was fortuitous here as it highlighted a difference between patients and healthy controls in the connectivity with the cerebellum, interesting differences in FC between the cerebellum and other regions have been found with different seeds. For example, in the present study a strong difference in FC between patients and healthy controls was found with the cerebellum using the mPFC as a seed. Further, RSFC analyses have indicated reduced FC between prefrontal/limbic regions and some cerebellar lobules [93]. A cerebellum seed-based connectivity study indicated higher positive FC between the cerebellum and DMN in MDD compared to healthy controls [101]. Decreased Crus I–DMN connectivity has been associated with young adult MDD [94,101]. Going forward, conducting more task-based FC analyses to explore the relationship between the hippocampus, cerebellum, prefrontal cortices, and DMN may provide valuable insight into memory and emotion dysfunction in people with MDD.

### 4.3. Limitations and Future Directions

Although our findings expand our current understanding of the neural underpinnings of MDD, it is important to also address the shortcomings of our study. First, there was a significant difference in the ages of our groups, and our sample size, while higher than the previous one from our group [32], remains modest compared to large-scale RSFC studies (e.g., [102,103]). Task-based FC studies on this scale have yet to be published. Thus, there is significant room for improvement, expansion, and fine-tuning of task-based FC research.

Second, we did not employ multi-echo fMRI acquisition, and the resting-state and task stimuli were relatively brief (10 min and <10 s, respectively). This might have led to a low signal-to-noise ratio. Prior work has shown that longer scan durations improve the reliability of FC estimates [104] and that multi-echo fMRI acquisition enhances signal recovery in areas prone to susceptibility artifacts, such as the OFC and medial temporal lobe [105,106]. Future studies might benefit from a multi-echo approach to improve data quality. A second potential analytical limitation is that while PPI is an effective method to assess task-based FC [107], it is possible that the subliminal presentation of the stimuli (33 ms each) was too brief to elicit reliable PPI effects. Because PPI relies on detecting task-related modulations in FC, such rapid stimulus-driven neural responses may not generate sufficiently strong interaction terms. More studies comparing seed-based vs. ICA analyses can help us determine when each approach is appropriate and which approach is superior.

Third, we opted for a seed-based analysis using only two seeds. It is possible that alternative seed regions that have previously been significantly activated by the prevention goal priming [29,30], such as the right OFC, precuneus, or PCC, may have been more sensitive and revealed different connectivity patterns. In addition, while seed-based analyses allow for more targeted hypothesis-testing, there is always a possibility of dominant FC patterns being excluded from the results. For example, there could be patterns among networks focused on cognition or visual perception that we did not find, because our seeds were informed by DMN and emotion-regulating network theories. Whole-brain FC analyses using the Selves Task may yield interesting findings that could further inform seed-based analyses.

Finally, due to the substantial difference in duration between our task conditions (less than 10 s for each stimulus of interest) and the resting-state scans (10 min), we decided not to perform any direct statistical comparison of RSFC and task-based FC. This choice was made to avoid unreliable estimates driven by duration mismatch. Future studies designed specifically to compare task-evoked and intrinsic connectivity using matched or balanced durations would help clarify the underlying mechanisms of each condition.

## 5. Conclusions

By comparing results from task-based and resting-state connectivity using a seed-based approach, we were able to demonstrate some spatial overlaps between brain regions that were weak at rest but more present during the task (hippocampus–cerebellum), or that were weak during the task and became more pronounced at rest (mPFC-DMN). Together, this suggests that using both resting state and task-based analyses can offer complementary insights into brain network function in MDD. More specifically, using the mPFC as a seed, we found a more constrained DMN FC pattern during the task, compared to rest. Using the hippocampus as a seed, which is rarely done in the existing literature, we were able to demonstrate a hippocampus–cerebellum relationship that was further emphasized with the task-based connectivity approach, therefore suggesting that this FC pattern between those two subcortical regions that are often ignored could potentially represent a biomarker that can inform future depression research. RSFC studies have laid a solid foundation for our understanding of brain function in psychiatric disorder, and future research on task-based FC may offer helpful insight on brain connections that can keep expanding our knowledge of how the brain functions during specific thoughts, actions, and episodes.

## Figures and Tables

**Table 1:** Participant Demographics.

	MDD (n = 23)	HC (n = 17)
Age (Mean ± SD)	43 ± 14	33 ± 12
Sex [Female: Frequency (%)]	14 (61%)	8 (47%)
White [Frequency (%)]	18 (78%)	7 (41%)
Black [Frequency (%)]	2 (9%)	4 (24%)
Asian [Frequency (%)]	0 (0%)	5 (29%)
Latino/Hispanic [Frequency (%)]	1 (4%)	1 (6%)
Multiple Races [Frequency (%)]	2 (9%)	0
MADRS Score (Mean ± SD)	27 ± 5	
HDRS Score (Mean ± SD)	20 ± 6	
Current Episode Length in Years (Mean)	1.88 ± 1.19	

MDD = major depressive disorder, HC = healthy control, SD = standard deviation, MADRS = Montgomery–Åsberg Depression Rating Scale, HDRS = Hamilton Depression Rating Scale.

**Table 2:** List of significant clusters for MDD > HC, MDD, and HC groups with RSFC using the mPFC as a seed.

Cluster Size	x	y	z	t-Value	Region *
MDD > HC					
2382	42	−50	−26	−4.87	rITG, rLOC, rTOFusC, rCerebellum Crus 1, rCerebellum 6
2313	18	0	68	−4.33	rPreCG, rSFG, rMidFG, rIFG, rSMA
1951	20	−20	4	−3.55	rInsC, rThalamus, rPallidum, rPutamen, rPP
MDD					
17,085	−4	54	−12	21.29	lFP, rFP, lSFG, ACC, lParaCG
3570	20	0	68	−13.48	lSFG
2952	50	−32	32	−10.77	laSMG
2580	−36	−38	32	−10.94	raSMG
2426	−8	−56	18	7.65	lpTFusC
1654	58	−6	−24	9.03	Precuneus
1292	−52	−62	28	8.96	rMTG
492	50	−58	22	8.37	rAG, rsLOC
379	46	−50	−8	−7.93	rITG, rLOC
153	−24	−36	−20	6.69	rHippoG
139	−38	−44	−38	−8.88	lAG
129	22	−32	−18	6.08	lITG
128	−20	−8	−16	6.34	lAmygdala
107	−40	−56	−4	−6.7	lCerebellum
53	38	36	34	−5.73	rFP
HC					
14,764	−8	56	−2	25.61	rFP, lFP, ACC, rParaCG, lParaCG
1143	2	−52	22	9.83	rMTG
1012	44	−38	44	−11.96	raSMG
767	−28	−56	34	−16.97	lSPL
473	−28	−28	−20	10.8	lHippocampus
368	10	−66	50	−11.69	lSPL
276	60	−4	−20	10.61	Precuneus
211	−26	−4	58	−8.5	lpTFusC
126	−22	−4	40	−7.71	lPostCG
59	−52	−32	−32	−7.98	lITG
55	−2	−82	4	−7.85	lIntraCalcC
51	−18	−42	78	−8.87	lSFG
49	58	−64	24	6.73	lCereb
39	56	−28	−4	7	PCC
37	18	−32	36	−7.25	rpMTG
34	−16	−56	−60	−7.49	rsLOC

Pink = positive FC. For MDD > HC contrasts, these regions had higher FC in the MDD group compared to HC. Blue = negative. For contrasts, these regions had higher FC in the HC group compared to the MDD group. * The region with the most voxels in each cluster is shown in all tables, excluding the seeds (mPFC, hippocampus). For results with multiple significant clusters, the five regions with the most voxels in the largest cluster are listed. For results with 5 or fewer clusters, the five regions with the most voxels are shown for each cluster. For results with one cluster, all regions are listed. Bilateral regions are shown for clusters in which both regions were among the top 3 regions in the cluster. l = left, r = right; a = anterior, o = occipital, p = posterior, s = superior; ACC = anterior cingulate cortex, AG = angular gyrus, Cerebellum 6 = cerebellum lobule VI, FP = frontal pole, HippoG = hippocampal gyrus, InsC = insular cortex, IntraCalcC = intracalcarine cortex, ITG = inferior temporal gyrus, LOC = lateral occipital cortex, MTG = middle temporal gyrus, PCC = posterior cingulate cortex, PP = planum polare, PreCG = precentral gyrus, SFG = superior frontal gyrus, SMG = supramarginal gyrus, SPL = superior parietal lobule, TFusC = temporal fusiform cortex, TOFusC = temporal occipital fusiform cortex.

**Table 3:** List of significant clusters for MDD > HC, MDD, and HC groups using the left hippocampus as a seed. Pink = positive FC, blue = negative FC.

Cluster Size	x	y	z	t−Value	Region
MDD > HC					
9858	−56	−54	−44	−5.53	lCerebellum Crus 2, lCerebellum Crus 1, rLG, rCerebellum Crus 1
5054	−40	48	10	−5.43	lFP, rFP, rOFC, lIns, lOFC
2481	−16	10	66	3.98	lSFG, lMidFG, lFP, lSMA, rSFG
MDD					
14,408	−20	−20	−14	19.02	Brainstem, lCerebellum 4/5, lTP, Vermis, precuneus
2147	18	6	62	−10.37	rSFG
2128	40	−44	40	−12.25	rpSMG
1559	28	48	26	−8.2	rFP
1521	−46	−48	54	−13.26	lpSMG
492	0	50	−14	7.28	MedFC
192	−36	44	16	−7.29	lFP, rFP
104	−54	−30	−32	−7.33	lpITG
48	−34	−8	50	−5.85	lPreCG
HC					
21,168	−26	−16	−18	18.5	lOFC, brainstem, rLG, rOFC, SubCalC
1381	8	−52	56	−9.84	precuneus
744	32	52	26	−9.52	rFP
219	50	−46	42	−7.52	rAG
154	20	14	62	−7.35	rSFG
112	−18	8	66	−7.74	lSFG
96	−8	−48	−28	7.06	lCereb
71	−58	−40	46	−8.5	lpSMG
62	66	−26	6	7.41	rpSTG

**Table 4:** List of significant clusters for MDD and HC groups using the mPFC as a seed, in the Prevention Match condition. Pink = positive FC, blue = negative FC.

Cluster Size	x	y	z	t-Value	Region
MDD					
7237	4	62	4	20.32	rFP, lFP, ACC, lParaCG, rParaCG
1488	−12	−54	24	8.49	Precuneus
294	−42	−66	28	6.94	lsLOC
206	64	−8	−24	9.02	rpMTG
163	46	−60	28	7.37	rsLOC
160	−20	36	48	7.2	lSFG
147	−54	6	−30	8.35	laMTG
114	44	8	−40	8.03	rTP
90	14	−66	62	−8.02	rsLOC
59	32	16	−16	7.76	rOFC
53	−12	−68	60	−7.63	lsLOC
33	26	−22	−14	7.56	rHippocampus
31	−18	−68	46	−6.5	lsLOC
31	32	20	−44	7.63	rTP
28	32	−48	60	−6.59	rSPL
25	−40	4	−44	7.42	lTP
25	4	−52	−48	6.69	rCerebellum
HC					
4685	6	60	−2	16.14	ACC, rFP, lFP, rParaCG, lParaCG
446	−6	−50	28	9.7	PCC
262	−14	38	46	10.69	lSFG
189	18	32	44	12.16	rSFG
187	32	32	−14	17.8	rOFC
137	−40	−60	26	9.12	lAG
64	−60	−6	−22	9.71	laMTG
37	−28	−54	44	−8.95	lSPL
19	32	−48	42	−7.47	rSPL
16	−32	−24	18	9.3	rAG
16	48	−58	28	7.39	lInsC
14	36	−38	38	−8.29	rpSMG
13	56	−48	−12	−9.4	rtoITG

**Table 5:** List of clusters showing significant differences for MDD > HC, and MDD group independently using the left hippocampus as a seed, in the Promotion + Prevention > Control contrast. Blue indicates negative FC.

Cluster Size	x	y	z	t-Value	Region
MDD > HC					
1991	−36	−68	−38	−5.73	lCerebellum 8, lCerebellum Crus 2, rCerebellum 8, lCerebellum, rCerebellum Crus 1, lCerebellum 6, lCerebellum 7b, lCerebellum 9, lOFusG, rCerebellum, 7b Vermis 9/8, lTOFusC, lLG, rCerebellum Crus 1, rCerebellum 6
MDD					
1447	4	56	20	−5.35	rParaCG, AC, rFP, SubCalC, lFP, rSFG, lSFG, lParaCG, lAccumbens, lCaudate

## Data Availability

Data are accessible on Open Neuro: https://openneuro.org/datasets/ds006731 (accessed on 29 September 2025).
